# An Overview of Asthma and COVID-19: Protective Factors Against SARS-COV-2 in Pediatric Patients

**DOI:** 10.3389/fped.2021.661206

**Published:** 2021-04-29

**Authors:** Maria Liuzzo Scorpo, Giuliana Ferrante, Stefania La Grutta

**Affiliations:** ^1^Department of Health Promotion, Mother and Child Care, Internal Medicine and Medical Specialties, University of Palermo, Palermo, Italy; ^2^Institute for Biomedical Research and Innovation, National Research Council, Palermo, Italy

**Keywords:** COVID-19, SARS-CoV-2, asthma, children, protective factor

## Abstract

Coronavirus disease 2019 (COVID-19) is a pandemic infectious disease caused by severe acute respiratory syndrome coronavirus SARS-COV-2. Aberrant innate immunity response and cytokine storm are responsible for the syndrome. Apparently, in asthmatic patients, the inadequate antiviral immune response and the tendency for asthma exacerbation evoked by common respiratory viruses could explain increased susceptibility to SARS-COV-2 infection. However, asthma has not been suggested to be a risk factor in COVID-19 patients. Therefore, in asthmatic patients some potential protective mechanisms against SARS-COV-2 have been hypothesized, like type 2 immune response, number of eosinophils, overproduction of mucus, and asthma treatment, along with behavioral factors not strictly related to asthma, such as social distancing, hygiene measures and wearing facemasks, that contribute to reduce the individual susceptibility to SARS-COV-2 infection. In this mini-review, we will describe the current literature regarding potential protective factors against COVID-19 in children with asthma based on the evidence available so far.

## Introduction

Coronaviruses are a family of single-stranded RiboNucleoid Acid (RNA) viruses able to infect humans, other mammals, and avian species. Human coronaviruses usually cause seasonal and mild respiratory tract infections which spread predominantly by droplets, aerosols and contact transmission, even though severe acute respiratory syndrome coronavirus (SARS-CoV), Middle East respiratory syndrome coronavirus (MERS-CoV) and SARS-CoV-2, which have appeared over the past 20 years, are considered highly pathogenic (1, 2). The infection caused by SARS-CoV-2, also recognized as Coronavirus Disease 2019 (COVID-19), began in Wuhan (China) and quickly spread all over the globe, so that this outbreak was proclaimed a pandemic on March 11, 2020, by the World Health Organization (3). COVID-19 presents with general and respiratory symptoms (fever associated with fatigue, myalgia, anorexia, dry cough, dyspnea), from mild to severe, which can be associated with a so-called “cytokine storm” (1, 2). Indeed, some patients show fast progression of the disease, ending in acute lung damage and multiple organ failure, whereas many infected people show mild or no symptoms. The median incubation period is approximately 5.1 days with 97.5% of symptoms becoming evident within 11.5 days (2). Early evidence from China and Italy indicated that children have less severe clinical manifestations than adults, with lower infection rates and reduced hospital admissions due to a lower incidence of severe disease and minimal mortality (4). Based on the available literature, it is believed that children could be spared from COVID-19 likely due to the highly expressed thymic repertoire and efficiently activated immune response against SARS-COV-2 (5). Out of the total cases, children accounted for only 1.2% in Italy, 5% in the USA and 2% in China (6). Clinical manifestations, laboratory tests and radiological findings in children with COVID-19 are described in Table 1. One severe complication of SARS-CoV-2 infection in children is a Kawasaki-like disease also known “multi-system inflammatory syndrome in children” (MIS-C). MIS-C derives from the unregulated release of proinflammatory cytokines and tissue damage–related enzymes that are responsible for multi-organ and/or multi-system failure (6). Because of generally low morbidity in children with COVID-19, it is difficult to ascertain the impact of SARS-CoV-2 on this population (7). Several factors have been hypothesized to offer an explanation on the low severity of the disease in the pediatric age group. First, seasonal coronaviruses may give a protective immune response toward SARS-CoV-2 (8). Second, children show low expression or function of the angiotensin-converting enzyme 2 (ACE 2) receptor (9). Third, in children there are “innate” B cells, called immune naïve cells, that respond to novel antigens, producing effective immune responses against the pathogen and possibly contributing to the lower pathogenicity of SARS-COV-2 in the pediatric age (10). Fourth, in the pediatric age immature B cells secrete anti-inflammatory cytokines such as IL-10, which may contribute to reducing the immune-mediated tissue damage (11). Moreover, the lower severity of COVID-19 in children with respect to adults could be explained by a stronger innate immune response and by the lack of co-morbid conditions in most subjects (6). Many risk factors such as diabetes, obesity and cardiovascular diseases are associated with COVID-19. Surprisingly, despite being a chronic respiratory disease, asthma would not seem a risk factor for COVID-19 either in childhood or in adulthood (1, 2, 12). In this mini-review, we will describe the current literature regarding protective factors against COVID-19 in children with asthma based on the evidence available at this time.

**Table 1:** Clinical manifestations, laboratory, and radiologic findings in children with COVID-19.

**Clinical manifestations**	**Laboratory test**	**Chest radiography**	**Computed tomography**	**Echocardiogram**	**Abdominal ultrasound**
Fever Myalgia Fatigue Nasal congestation and rhinorrhea Sore throat Cough Dyspnea Nausea/Vomit Diarrhea	Neutrophilia Lymphopenia Thrombocytopenia High levels of erythrocyte sedimentation rate, C-reactive protein, procalcitonin, ferritin, fibrinogen, liver enzymes, D-dimer, troponin, creatine and blood urea nitrogen	Peribronchial cuffing Perihilar interstitial thickening Atelectasis Small pleural effusions	Pulmonary groundglass opacities	Myocardial dysfuction Pericardial effusion Coronary artery aneurysms	Bowel wall thickening Lymph nodes enlargement

## Pathogenesis of COVID-19

SARS-COV-2 necessitates two proteins for entry into the host cell. The virus attaches to the ACE 2 receptor; subsequently, the host trans-membrane protease serine 2 (TMPRSS2) splits up the spike protein, expressed on the viral envelope, into two segments, allowing fusion of SARS-COV-2 to the cellular membrane and its penetration into the cell. The binding of SARS-CoV-2 to ACE 2 receptors produces a marked down-regulation of these molecules, whose protective effects on the human body have been recognized. The loss of such protective effects could result in interstitial fibrosis, endothelial dysfunction, marked inflammation, oxidative stress and enhanced coagulation (13). In fact, after its entry into the host cells, the activated innate immune response prompts release of pro-inflammatory cytokines, which recruit effector cells like neutrophils, macrophages, etc. In the context of the adaptive immune response, antigen-presenting cells (APCs) present viral antigens to T cells, eliciting differentiation from immature cells to mature cells (Cytotoxic T cells and Natural Killer cells) that might contribute to killing virus-infected cells. In particular, once the virus is inside the cells, viral peptides are presented through Class I Major Histocompatibility Complex (MHC) proteins to CD8+ cytotoxic T cells (CD8+ T cells). Activated CD8+ T cells clonally expand and differentiate into virus-specific effector and memory T cells. The virus-infected cells are killed by CD8+ T cells. Dendritic cells and macrophages (APCs) recognize the virus and its particles and present them to CD4+ T cells through MHC-Class-II molecules. The viruses can activate B lymphocytes that can interact with CD4+ T cells. In the first week following symptoms, the primary antibody response determines an increased amount of virus-specific IgM followed by production of virus-specific IgGs (14). If the adaptive immune responses are insufficient, innate immune responses can be reinforced through a cytokine storm that is responsible for severe multi-organ damage (1, 2). In case of low-dose virus infection, efficient T- and B-cells responses and neutralizing antibodies could lead to rapid viral clearance. By contrast, high-dose virus exposure may account for severe disease and delayed viral clearance. This can be attributed to lymphopenia, which determines inadequate T- and B-cells responses, eventually followed by a cytokine storm and multi-organ failure (14).

## Pathogenesis of Asthma

Asthma is a respiratory disease characterized by chronic inflammation of the airways with bronchial hyper-responsiveness to several stimuli, mucus overproduction, recurrent episodes of wheezing, respiratory distress, and cough, associated with reversible airway obstruction. Asthma is one of the most common chronic diseases worldwide, affecting more than 300 million individuals, and the incidence is growing, particularly in developed countries (15). Asthma remains one of the highest causes for school absence and hospital admissions, imposing a high socioeconomic burden, and impairing quality of life of children and their families (16, 17). Allergic asthma is the most common type, where exposure to allergens in sensitized subjects specifically triggers a type 2 (T2) inflammatory response (1, 2). Because of the tendency for disease exacerbation elicited by common respiratory viruses including Rhinovirus, Respiratory Syncytial Virus, Influenza virus, Parainfluenza virus, Adenovirus, human Bocavirus, and Coronaviruses (18) and a deficient antiviral immune response that is evident in asthmatic patients (2, 19, 20), the latter should potentially have increased vulnerability to SARS-COV-2 infection. This could be sustained by the deficient type I Interferon (IFN) responses observed in patients with severe asthma. In particular, activated T helper (Th) 2 cells secrete Interleukin (IL)-4/13 and IL-5, which are responsible for activation of other inflammatory cells, including eosinophils (21). Eosinophils organize the immune response against respiratory viruses, releasing cytotoxic proteins and nitric oxide, producing type 1 cytokines (IL-12 and IFN gamma) and recruiting CD8+ T cells. Eosinophils also contribute to clearing the viral load, insuring recovery from infection. Moreover, IL-4/13s increase IgE production by B cells, further impairing the activation of innate immune response. Increased expression of T2 cytokines, TGF-β and Suppressor Of Cytokine Signaling (SOCS) 1/3 negatively regulates type I IFN production, further increasing the risk of viral infections (22, 23). However, some studies have shown that asthma is not a risk factor in patients with COVID-19 (3). In the study by Zhang et al., involving 140 community-infected COVID-19 subjects, asthma was not reported by any of the patients (24). Similarly, Dong et al., in a case series of 2,135 pediatric patients with COVID-19, did not report any case of asthma (25). In the Confidence study, which included 100 pediatric patients with COVID-19, chronic respiratory diseases did not appear as risk factors (26).

## Protective Factors Against SARS-COV-2 Infection in Children With Asthma

**Figure 1:**
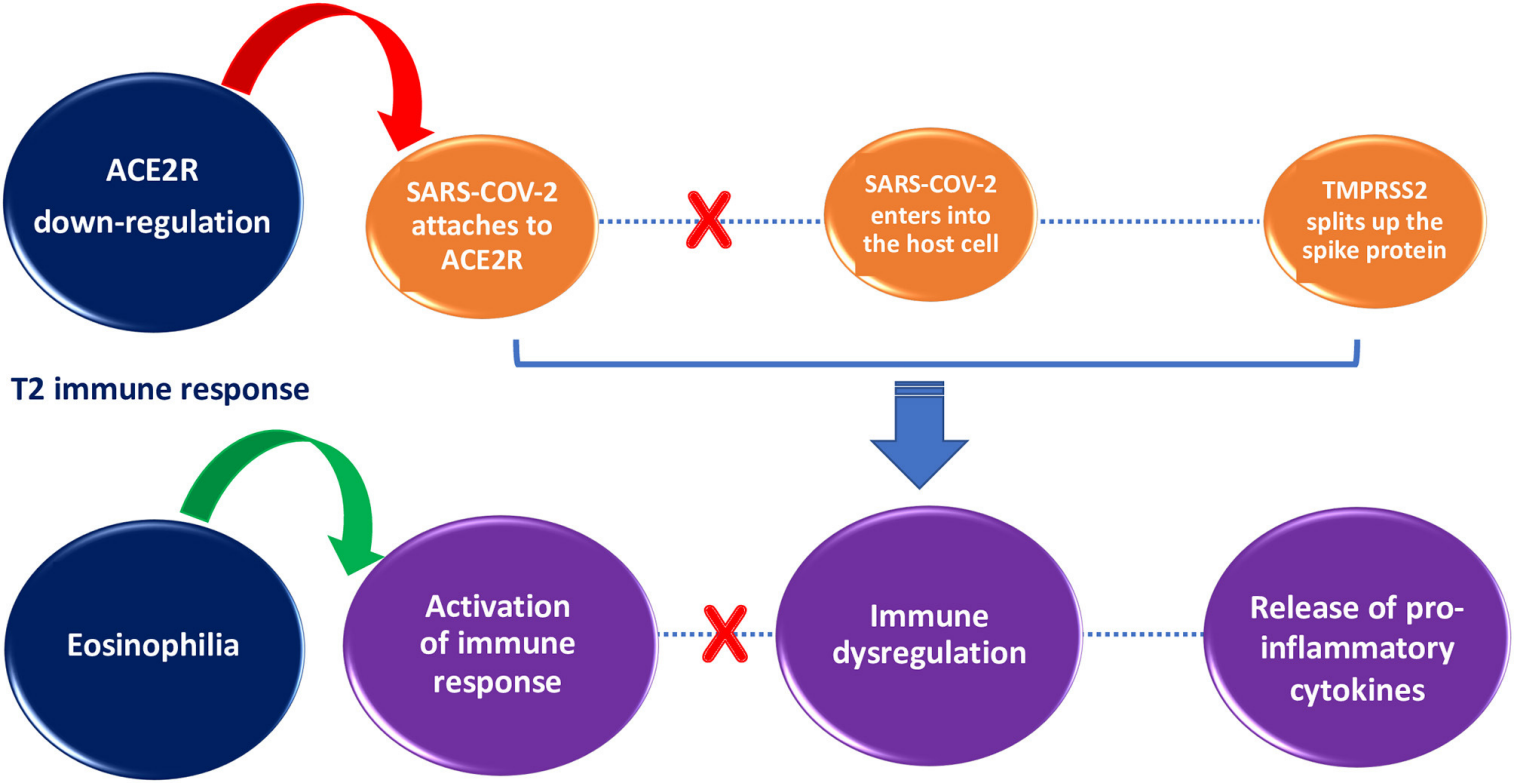
Impact of T2 immune response on SARS-COV-2 infection susceptibility and severity. T2 immune response inhibits SARS-COV-2 entry into the cell through ACE2R down-regulation (red arrow) and helps to clear the viral load through activation of immune response orchestrated by eosinophils (green arrow).

Some protective factors against SARS-COV-2 infection have been hypothesized in patients with allergic asthma, such as T2 immune response (Figure 1), overproduction of mucus, and asthma treatment (27–30).

### Type 2 Immune Response

The T2 immune response in asthmatic patients might counteract the COVID-19 cytokine storm (2). According to Kimura et al., in airway epithelial cells of patients with allergic asthma ACE 2 is reduced and TMPRSS2 is increased thanks to IL-13 exposure. In addition, patients with activated Th2 immune responses showed decreased expression of ACE 2 in airway epithelial cells, inversely correlated with T2 cytokine levels and Th2 signature molecule expression (31). In the study by Sajuthi et al., nasal airway transcriptome and network co-expression analysis were used to detect cellular and transcriptional factors of ACE 2 and TMPRSS2 genes in a cohort of 695 subjects with asthma and healthy controls between 8 and 21 years of age. They found that Th2 inflammation had a major role in ACE 2 down-regulation as well as in TMPRSS2 up-regulation. The study also showed that ACE 2 expression was marked only in secretory cells and ciliated cells, whereas TMPRSS2 was expressed in all epithelial cell types. Overall, these results suggest that Th2 immune responses may be a protective factor against SARS-COV-2 infection by causing ACE 2 down-regulation (32). Additionally, since eosinopenia has been observed in COVID-19 patients, the increased number of eosinophils in asthmatic patients could have a protective role against SARS-COV-2 (27). Though the relationship between eosinophil levels and COVID-19 is still not clear, during the pandemic it is important to monitor eosinophil counts and the clinical course of COVID-19 in patients with asthma treated with biological drugs responsible for decreased eosinophil levels (33). Indeed, anti-IL-5 biologics such as mepolizumab reduce eosinophils, and apparently could increase susceptibility to viral infections (5).

### Overproduction of Mucus

Mucus hypersecretion could be recognized as another hypothetical protective factor against COVID-19 because it acts as the first line of defense against infection, thereby preventing SARS-COV-2 from reaching the distal airways and entry into the alveolar type 2 cells, which predominantly express ACE 2 in the lung. In asthma there is increased expression of MUC5AC (34), which has been proved to give protection against influenza infection in a murine model (35). However, not all patients with asthma show mucus overproduction; thus, mucus hyperproduction may only give protection in some patients. Moreover, it should be recognized that other respiratory diseases characterized by mucus hypersecretion like COPD showed poor outcomes from SARS-COV-2 infection (29).

### Asthma Treatment

Inhaled corticosteroids (ICS) are the first line treatment of asthma. One study hypothesized that ICS could increase anti-viral immunity in treated patients (27). There is also evidence that ICS may down-regulate both ACE 2 and TMPRSS2 expression, thereby decreasing binding of SARS-COV-2 to receptors on the airway epithelium cells (12, 20, 30, 36). Moreover, ICS suppress virus replication and cytokine production (28). Matsuyama et al. have demonstrated that ciclesonide blocks SARS-COV-2 replication *in vitro* and inhibits its cytopathic activity, reducing the severity of the disease. Additionally, the combination of formoterol, glycopyrronium and budesonide has been shown to inhibit seasonal coronavirus replication and cytokine production (37). Peters et al. studied ACE 2 and TMPRSS2 gene expression in sputum cells of 330 patients with asthma and 79 healthy controls, finding that use of ICS was associated with lower expression of ACE 2 and TMPRSS2 after adjustment for asthma severity. Therefore, these data provided further explanation about factors that may be responsible for the low prevalence of asthma among COVID-19 patients. However, it should be considered that though ACE 2 receptors are particularly expressed in type 2 pneumocytes and sputum, this may not reflect ACE 2 receptors levels in the lower airways. Further, since the level of deposition of many ICS is low in peripheral airways, they might not affect ACE 2 expression in type 2 pneumocytes (12). Moreover, even though corticosteroids are not considered a fundamental therapy for lung injury in COVID-19 patients, it has been hypothesized that they may be beneficial to suppress the cytokine storm typically associated with the advanced phase of the disease (27).

Allergen immunotherapy (AIT) suppresses T2 immune responses and controls allergic inflammation by stimulating T regulator cell responses and preventing tissue homing and degranulation of mast cells, basophils and eosinophils (38, 39). Therefore, it could be supposed that AIT might play a role in preventing a cytokine storm (2, 27).

In recent studies, the monoclonal antibody against human IgE Omalizumab has been suggested to have a potential effect on antiviral responses by reducing susceptibility to respiratory virus infections (2, 27). The recently approved biologic drug, Dupilumab, a human IgG4 antibody anti-IL-4 receptor (IL-4R) α-subunit that blocks IL-4R signaling induced by both IL-4 and IL-13, may also exert a protective role as it induces ACE 2 down-regulation (33, 40).

## Discussion

In spite of inadequate antiviral immune responses and vulnerability to acute exacerbations due to viral infections, there is evidence that asthmatics have reduced susceptibility to SARS-COV-2 infection (2). Overall, this might be ascribed to specific asthma-related factors such as T2 responses, overproduction of mucus, asthma treatment along with behavioral factors not strictly related to asthma, such as social distancing, hygiene measures and wearing facemasks, helping to reduce individual susceptibility to SARS-COV-2 infection (3, 27). Social distancing and facemasks may decrease viral transmission from person to person (41). Evidence suggests that adherence to preventive measures is influenced by the perceived risk of infection. In a cross-sectional study on adolescents with chronic diseases, including asthma, 60% respondents reported high adherence to preventive measures such as hand-washing, avoiding group gatherings, reducing use of public transportation and avoiding public places. The rate of adherence to preventive measures was similar for respondents with and without chronic diseases (42). However, evidence on young children is lacking. With regard to facemasks, their use has been shown to reduce transmission of coronaviruses and influenza viruses by blocking the release of virions into the air (43). This is particularly relevant in children with asthma given that respiratory viruses are main drivers of exacerbations (44). However, during the first wave of the pandemic, children with asthma did not show severe COVID-19 infection, regardless of asthma severity and control (45). Moreover, improved childhood asthma outcomes were observed, namely reduced acute attacks, emergency department visits, and hospitalizations as well as improved scores in asthma control measures and lung function. These findings might be due to reduced exposure to outdoor asthma triggers, such as seasonal allergens and air pollutants, and increased treatment adherence (46–48).

Based on the available literature, COVID-19 outcomes vary from mild to severe clinical manifestations and this may reflect different airway levels of ACE 2 and TMPRSS2, which are indispensable for virus entry into the host cell. Several authors have hypothesized that in children with asthma T2 immune response may lead to ACE 2 down-regulation and TMPRSS2 up-regulation, thereby reducing the risk of illness due to SARS-COV-2 (3, 18, 27). Additionally, a recent study involving children and adults with asthma from three different cohorts demonstrated that ACE 2 receptor levels were lower in patients with allergic sensitization, whereas no association was found in patients with non-atopic asthma (3, 27). Therefore, decreased ACE 2 expression may have an important role along with several other factors in reducing COVID-19 severity in patients with allergic asthma (3, 18, 27). As for blood eosinophil counts, reports from China suggested that a worse prognosis of COVID-19 can be hypothesized when patients show severe eosinopenia and lymphopenia. In addition, it has been hypothesized that SARS-CoV-2 infection could be related to low eosinophil levels in peripheral blood and that high blood eosinophil counts in children with asthma could be a protective factor against SARS-CoV-2. Nonetheless, others studies did not show low eosinophil counts in patients with severe COVID-19 (49, 50). Though the relationship between eosinophil levels and COVID-19 is still uncertain, monitoring level of eosinophils in asthma patients has been suggested, especially in those treated with biological drugs that provoke a reduction of eosinophils in peripheral blood (3, 27). In addition, some authors hypothesize that cytokines and chemokines involved in T2 immune responses, like IL-13, IL-9 and Macrophage Inflammatory Protein-1 alpha and beta, might counteract the pool of proinflammatory cytokines involved in the pathogenesis of COVID-19. Since few data are available, other studies are needed (2).

Overproduction of mucus in asthma has also been suggested to have a protective role, since mucus, rich in mucin glycoproteins, acts as the first line of defense against viruses; however, further studies are needed to confirm this hypothesis (29).

With regard to asthma treatment, evidence suggests that ICS are associated with decreased ACE 2 and TMPRSS2 gene expression and that taking ICS may be beneficial in treating coronavirus infections (12). In particular, a study *in vitro* showed that budesonide can inhibit HCoV-229E replication and cytokine release, and demonstrated that SARS-CoV-2 RNA replication and its cytopathic effects are blocked by ciclesonide, suggesting a protective role against this virus (37). However, given the low level of many ICS deposition in peripheral airways, they might not affect ACE 2 expression in type 2 pneumocytes. Furthermore, ICS may increase antiviral immunity which is typically inhibited by T2 immune responses (12, 20, 27, 30, 37). Studies *in vitro* demonstrated that glycopyrronium has an inhibitory effect on seasonal coronavirus replications and cytokine production. These effects are reinforced when glycopyrronium is used in combination with formoterol and budesonide *in vitro* (27, 37). Nonetheless, glycopirronium is not routinely used in children and more studies are needed to better understand if asthma treatment could really be considered protective against SARS-COV-2 infection (27, 51).

Poor evidence is available about the role of AIT and biologic drugs; it is possible that AIT is protective against the cytokine storm occurring in severe COVID-19 by inducing a state of immune tolerance. As for biologic drugs, some studies hypothesized that Omalizumab may have a protective role against viral infections that induce asthma exacerbations and that Dupilumab may have a role against SARS-COV-2 as it could reduce expression of ACE 2 (2, 27, 33). Therefore, in light of the abovementioned data and according to the Global Initiative for Asthma Guidelines (GINA) recommendations, children with asthma should continue their treatment to prevent asthma exacerbations due to SARS-COV-2 infection (1, 27, 52), undergoing pulmonary function tests when needed to guide management (53).

In summary, based on the available literature, whether asthma could really be considered a protective condition against SARS-COV-2 infection in children is still not clear. Therefore, further studies are required to clarify the impact of asthma on COVID-19 susceptibility and severity, especially in pediatric population where the available evidence is very limited. Nonetheless, parents and caregivers can be reassured that severe COVID-19 infection is generally rare in children, irrespective of their having asthma. Additionally, it should be pointed out that, even in countries with a high number of COVID-19 infections, the incidence of COVID-19 did not increase with the reopening of schools and outbreaks in schools occurred uncommonly, especially when precautions to control virus transmission were taken (54).

## Author Contributions

ML and SL: conceptualization. ML and GF: writing original draft. GF and SL: review and editing.

## Conflict of Interest

The authors declare that the research was conducted in the absence of any commercial or financial relationships that could be construed as a potential conflict of interest.
